# Older Adults’ Beliefs, Knowledge and Preferences for Achieving Healthy Vitamin D Status: A Narrative Review

**DOI:** 10.3390/geriatrics3020026

**Published:** 2018-05-30

**Authors:** Tatiana Christides

**Affiliations:** Department of Life & Sports Sciences, Faculty of Engineering & Science, University of Greenwich, Medway Campus, Chatham-Maritime, Kent ME4 4TB, UK; t.christides@greenwich.ac.uk; Tel.: +44-(0)208-331-8427

**Keywords:** vitamin D, older adults, knowledge, preferences, beliefs, practices, autonomy, falls, mobility

## Abstract

Autonomy and mobility are, in many cases, key elements underlying positive ageing. Vitamin D (vitD) is essential to maintaining musculoskeletal health and hence mobility; ensuring adequate vitD status is important in positive ageing. However, vitD deficiency persists in ~10–30% of older adults in the Western world. The aim of this review was to explore older adult vitD beliefs, knowledge and preferences, in order to identify means to prevent vitD deficiency respectful of older peoples’ autonomy. Academic search-engines were used to explore the research literature with the keywords: vitamin D; older adults; preferences; knowledge; practices; beliefs. 22 recent studies were identified; although the majority of older people knew of vitD, knowledge about increased fall risk secondary to deficiency was limited and knowledge did not always correlate with adequate intake or status. There was evidence of confusion regarding vitD food sources, sun-exposure and health benefits, and although General Practitioners were trusted information sources they often did not discuss vitD with patients. Preferences varied significantly depending on geographic location, ethnicity, socioeconomic status, education and cultural factors; overall, older people wanted more clear information about vitD. In conclusion, older people have a relatively high awareness of vitD, however, knowledge may be inaccurate and low in those most at risk, and knowledge of deficiency-associated fall risk is under-recognised. Furthermore, studies specifically addressing older adult preferences are lacking; an understanding of preferences, integrated into public health guidelines and implementation strategies, is key not only to decreasing the risk of vitamin D deficiency but also to enabling autonomy in older adults.

## 1. Introduction

Mobility and its associated independence are key to health-related quality of life and positive ageing in many cases [1,2,3]. Mobility provides independence and autonomy. These are important factors in enabling positive ageing, defined by the Centre for Positive Ageing in collaboration with an older adult advisory group, as the ability to “live life’s changes… in a productive, active and fulfilling manner” supported by research that promotes “a person’s sense of independence, dignity, well-being, [and] good health” [4]. Mobility and the benefits derived from it are enabled and sustained by good musculoskeletal health. Sufficient vitamin D body status, defined as blood levels of 25(OH)cholecalciferol (the circulating form of the vitamin that most accurately reflects body status) ≥50 nmol/L [5] is necessary for musculoskeletal health [5,6,7,8,9]. This is because vitamin D maintains calcium and phosphorus homeostasis that is essential both for bone mineralization, and neuromuscular function and muscle strength [5,6,7,8,9]. Vitamin D is certainly only one factor, amongst many, required for maintaining mobility. For example, other factors include management of polypharmacy, and exercise programmes [10,11], but nonetheless without adequate vitamin D body levels to maintain musculoskeletal health, the risk of falls and subsequent injury increases, and muscular strength is compromised, thus potentially decreasing mobility and independence [10,11]. Consequently, ensuring adequate vitamin D status in older people to maintain musculoskeletal health is an identified public health goal as highlighted by several recent reports [5,6,8].

Vitamin D can be obtained through the diet from a limited number of foods such as oily fish, eggs and dairy products. Vitamin D may also be obtained from sunlight-derived ultraviolet irradiation (UVR) acting on vitamin D precursors in the skin leading to cutaneous synthesis [5]. However, UVR of the needed wavelength for cutaneous vitamin D production is dependent on latitude, and principally occurs during the spring, summer and early autumn months in temperate zones, and then primarily between 11:00 a.m. and 3:00 p.m. [5]. In London (United Kingdom), for example, cutaneous synthesis occurs between late March through early October [5]. Public Health England (PHE) recommends that older adults, defined as people 65 years old and over (65 y/o+), consider taking a daily vitamin D supplement containing 10 μcg vitamin D per day, as it is difficult to meet recommended intakes through food or sun-induced cutaneous vitamin D synthesis [8]. The Chief Medical Officers of England, Scotland and Wales sent out letters in 2012 to General Practitioners (GPs), practice nurses and community pharmacists, advising that older adults, amongst other “at risk” groups, be made aware of the recommendation to take a daily supplement [12]. Despite these recommendations, poor vitamin D status in older adults persists in a significant minority of people. 10–30% of people over the age of 65 have vitamin D blood levels <30 nmol/L that is defined as “at risk for deficiency” by the United Kingdom(UK) Department of Health (DoH) (30–50 nmol/L is defined as “insufficient”); this is moderated by sociodemographic characteristics with older adults in care homes being at greatest risk [5,6,13,14]. Vitamin D dietary intake in older adults in the UK is consistently low at approximately 2.5–3 μcg/day (the UK reference nutrient intake (RNI), for people 65 y/o+, is 10 μcg/day) [5,6,8,13], and though not well studied, evidence suggests that uptake of supplements is low [6]. These factors, combined with inadequate sun exposure, most likely explain why vitamin D deficiency persists in this age group [5,6] .Unfortunately, facilitators and obstacles to supplement use have not been well studied in any age group, and in particular there is little data available specifically related to older adults; furthermore, older adult preferences, for both how they would wish to ensure adequate vitamin D status and how they believe vitamin D awareness should be increased, are poorly explored research questions [5,6].

The purpose of this narrative review is to evaluate the evidence in the scientific literature that explores knowledge, beliefs, practices and experiences of older adults with regards to vitamin D, and their preferences for sustaining healthy vitamin D body status, with the goal of identifying means to prevent vitamin D deficiency that are respectful of older people’s preferences and autonomy.

## 2. Methods

**Figure 1:**
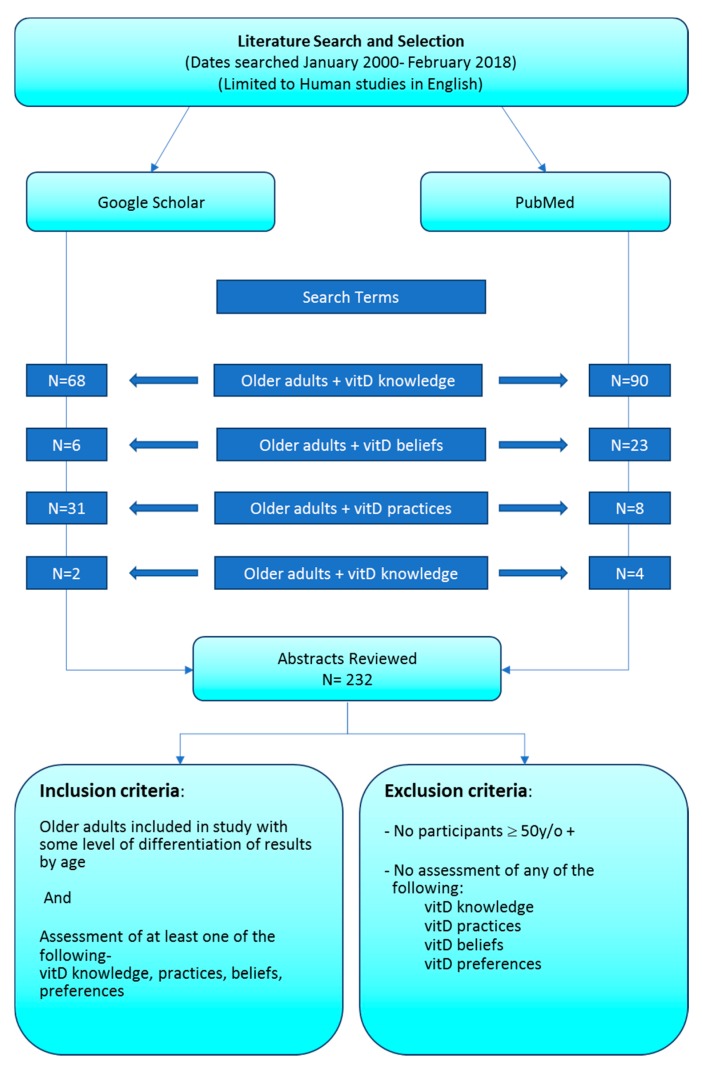
Literature review search engines, terms, study exclusion and inclusion criteria.

Google Scholar and PubMed were used as sources to explore the research literature with the keywords: older adults + vitamin D: preferences; knowledge; practices; and beliefs. Studies were limited to the last 18 years (January 2000–February 2018) to reflect current trends; one survey from a charity was included otherwise all considered publications were published in peer reviewed journals. Sources were searched starting December 1st 2017 and finishing 2nd May 2018. Study types included qualitative studies, quantitative survey-based studies and/or studies with vitamin D-related blood work. Studies were selected for analysis and discussion on the basis that they evaluated older adults’ knowledge, beliefs, preferences and experience related specifically to Vitamin D; 22 studies were selected for review. Please see Figure 1 for a flow chart of the literature search and selection methods. As this was not a systematic review search terms were narrowly defined and the author does not wish to suggest that this was an exhaustively comprehensive review.

## 3. Results

### 3.1. Older Adult Knowledge, Beliefs, Practices and Preferences Related to Vitamin D—Numbers of Studies

There have been a large number of studies exploring knowledge and beliefs about vitamin D. For example, a search on Google scholar using the words “vitamin D” + “knowledge” returned 2.4 million results (May 2018); specifying “older adults” decreased this to 22,000 studies. The scope may be further decreased by searching only within this millennium, and further specifying older adults + “vitamin D knowledge”, “vitamin D preferences”, etc. as was carried out in this review. Studies with only older adults in comparison with studies of mixed ages were fewer −9 versus 13, respectively, in this review. The author could not identify any studies with the sole specific research question of identifying older adult preferences for obtaining vitamin D; however, this topic did emerge in various studies exploring beliefs and practices related to vitamin D.

Table 1 summarises the 22 studies presented in this review; studies are listed in the order in which they are presented in results.

### 3.2. Mixed-Age Population Studies

One of the most recent, and the largest identified study, explored “What Do People Know and Believe about Vitamin D?” [15]. It was conducted in France; vitamin D knowledge and beliefs were measured in a subset of people participating in a large online study (the NutriNet-Sante Study) evaluating associations between nutrition and health [15]. Amongst the approximately 60,000 participants, approximately 38%—23,000 adults—were over the age of 55, (herein referred to as older adults for the purposes of this study); no further age breakdown was provided. Vitamin D knowledge and beliefs were measured using a validated online questionnaire, and in a subset sample of participant blood samples were taken and vitamin D levels measured. Older adults had the highest levels of awareness of vitamin D compared with all other age groups, 94.5% versus an average of 92%. Older adults also cited physicians, newspapers and radio as their main sources of knowledge about vitamin D more often than younger participants. Older adults were more aware that vitamin D had a role in bone health and rickets compared with all age groups and were also better informed about dietary sources of the vitamin, although approximately 20% of older adults were unaware of the association between musculoskeletal health and vitamin D, and approximately 33% of older participants incorrectly identified dietary sources of vitamin D—for example, stating that vegetables were a vitamin D food source. However, participants who obtained information from physicians had better knowledge both of vitamin D health effects and dietary sources. No questions were asked about the role of vitamin D in muscle health in the context of fall prevention, nor were participants asked about their preferences for obtaining vitamin D. Interestingly, knowledge about vitamin D did not correlate with measured vitamin D status, and only 16% of participants with measured low vitamin D blood levels were concerned about their vitamin D status—this finding was not reported by age [15].

Several mixed-age population studies have been conducted in Australia evaluating vitamin D knowledge, beliefs and/or practices. The largest of these (*n* = 12,513), a mixed methods study using questionnaires, 24 h dietary recall for nutritional assessment, interviews, and blood sampling of 25(OH)cholecalciferol, was interested in predictors of vitamin D/ vitamin D containing supplement use amongst the general population [16]. Approximately 33% of participants were 55 y/o+ including 25% 71 y/o+. Vitamin D/ vitamin D containing supplement use increased with age, although even in the 71 y/o+ group only one in four people were using supplements, and amongst men only 15% were taking supplements. Interestingly, only 4% of participants were using vitamin D-alone supplements as recommended by the government, rather most people received varying amounts of vitamin D as part of multivitamin preparations. Higher educational attainment and socio-economic status were associated with supplement use, as was poorer self-assessed health. Use of supplements was associated with higher serum levels of 25(OH)cholecalciferol and vitamin D sufficiency [16].

Another study carried out in Australia looked at knowledge about vitamin D in relation to sun-related behaviours. This was a cross-sectional study of 2876 people including approximately 300 adults over the age of 50 (~9.5% of the study population) using an online survey delivered questionnaire [17]. 69% of participants had some knowledge of vitamin D and this was higher in older adults; however, the majority of participants incorrectly identified food sources of the vitamin, and correct knowledge about the amount of time that they would need to spend in the sun to obtain vitamin D was very limited—this did not differ by age [17]. Furthermore, only 10% of adults over 50, which would include the “at risk” group of people over 65 y/o, were concerned that they might have low vitamin D status. Almost half of participants had heard about vitamin D from various media outlets including newspapers, radio and the internet, and only 1 in 5 participants identified “professionals” (such as healthcare workers) as sources of knowledge about vitamin D.

A further Australian cross-sectional mixed-age population study (*n* = 2000; 40 y/o+ *n* = 1319) conducted by phone interview found that approximately 60% of participants were aware of at least one health benefit of vitamin D, although this was highest in adults 40 y/o+, and less than 10% of participants knew the amount of time they would need to spend in the sun to obtain adequate levels vitamin D [18] this finding was not reported by age. Information sources and preferences were not assessed in this study.

Bonesvki et al. carried out a small qualitative study (*n* = 52, including 9 people aged 65 y/o+ in the community, and 9 people aged 65 y/o+ in residential care homes; ~28% of the study population) in Australia, using the consolidated criteria for reporting qualitative research methodology and two health behaviour models for analysis of findings [19]. They evaluated the interactions between public health guidelines for sun exposure to avoid skin cancer versus the need for sun exposure for adequate vitamin D synthesis [19]. The majority of participants had heard of vitamin D, but many could not name its health effects, nor did they know the amount of sun exposure needed for cutaneous synthesis to provide adequate vitamin D status. Participants were much more aware of the guidelines about avoiding the sun to prevent skin cancer in comparison with sun exposure needed for vitamin D cutaneous synthesis. Perhaps not surprisingly, participants were also more concerned about skin cancer versus vitamin D deficiency risks—“It’s [skin cancer] deadly, and you die a lot quicker from cancer than you can from vitamin D deficiency...” [19]. This study did not explicitly explore older adult preferences for obtaining vitamin D, but within the context of “overcoming barriers” to address vitamin D deficiency, the majority of participants suggested increasing “incidental sun exposure” such as eating outdoors, or walking to a farther parking space, rather than food fortification or supplementation. Both the media and healthcare professionals were proposed as good sources for increasing awareness of vitamin D, with older adults specifically identifying physicians and community pharmacists as valuable information sources, but participants also stated that discussions about vitamin D with GPs were often limited. An important insight from this study was that participants felt the guidelines about vitamin D needed to be “simple”, and that how to achieve the balance of sun exposure needed to obtain vitamin D without increasing cancer risk needed to be clearly explained.

A large study (*n* = 4127) conducted in the USA explored the interaction between vitamin D beliefs and skin cancer risk-related activities. This was a cross-sectional retrospective study using online survey data derived from a larger study [20]. Approximately 20% of participants were 55–64 y/o, and 20% were 65 y/o+. Older adults were more likely to believe that diet and vitamins would provide adequate levels of vitamin D, whilst they were also more likely to protect their skin from the sun.

In Hong Kong a cross-sectional telephone- interview study explored knowledge and beliefs amongst a group of middle aged and older women; researchers were interested in understanding cultural and population specific behaviours related to vitamin D amongst Chinese women [21]. There were 547 participants of whom 31.5% were 65 y/o+. Unusually, further information about age stratification was provided—21% of participants were 65–74 y/o, 9% 75–84 y/o, and 1.5% ≥ 85 y/o, matching trends of older adult participation in research studies in which as age increases rates of participation decrease (for example, see [37]). In comparison with the studies previously discussed, in this study as age increased knowledge of health effects of vitamin D and food sources decreased. However, even amongst participants that had knowledge of vitamin D, only a minority knew of its effects on the musculoskeletal system; knowledge about the effects of vitamin D on falls was not assessed. In this study the majority of participants did not enjoy being out in the sun and this was not moderated by knowledge about vitamin D and sun exposure; indeed, subjects who had spent less than 1 h in the sun (previous week) were more likely to know that vitamin D could be obtained from sunlight (76.4% vs. 23.6% knowledge rate) [21].

Interestingly, the group that most enjoyed being in the sun were women ≥85 y/o, the same group with the lowest knowledge about vitamin D. All groups had poor knowledge about food sources of the vitamin, and the amount of time required to be in the sun in order to make adequate vitamin D.

In Kuwait a cross-sectional study using a structured questionnaire was conducted in 200 randomly selected vitamin D deficient patients undergoing treatment in multiple medical centres; 26% of participants were 60 y/o+; as age increased blood levels of vitamin D fell (the average level for people 70 y/o+ met clinically defined criteria for severe vitamin D deficiency) [22]. Approximately half of the patients were unaware of the association of low vitamin D status with musculoskeletal pain; no participants discussed the association of low vitamin D status with falls. 85% were aware that sunshine was a source of vitamin D though more precise knowledge about vitamin D and sunlight was not explored. Results were not presented by age.

A small qualitative study on knowledge and attitudes towards vitamin D was conducted in Saudi Arabia; there were 22 participants of whom 82% were 49 y/o+, and participants had semi-structured interviews subsequently analysed using thematic analysis [23]. Participants overall had good knowledge about vitamin D and its effects on bones, and the majority were also aware of the relationship between vitamin D and sun exposure although lacking in specific information about needed exposure time. Similarly, with several other studies, however, knowledge about dietary sources was low and, in some instances, incorrect dietary sources of vitamin D, such as fruit and vegetables, were identified. Attitudes towards sun exposure varied, and the high temperatures of the area were cited as a disincentive towards being outside during peak periods for cutaneous vitamin D synthesis.

A number of mixed-age populations studies looking at vitamin D knowledge, beliefs and practices have been carried in the UK.

A mixed-age population anonymous online survey was conducted by Mulhern et al.; results were presented at a Nutrition Society conference proceeding [24]. Investigators assessed awareness and knowledge of new dietary vitamin D guidelines proposed by the Scientific Advisory Committee of Nutrition (SACN [5]); 1320 individuals completed the questionnaire, the majority of whom lived in Northern Ireland. Approximately 50% of participants were young adults (18–34 y/o); further information on age range or stratification was not provided. Although the vast majority of participants had heard of vitamin D, only ~ one fourth (23.7%) were aware of the new guidelines, and amongst the people that had heard of the new guidelines, only 40%—i.e. about 1 in 10 people in the study—actually new the correct recommended intake of 10 μcg/day.

Another mixed-age population cross-sectional study was carried out in North West England amongst “at risk” groups (*n* = 221, average age 35, no further age categories were specified though the age range included people 65 y/o+ [25]. A validated questionnaire was used to measure knowledge about vitamin D including food sources, sun exposure, and health effects of deficiency. Approximately 30% of people had not heard of vitamin D; older participants had lower knowledge of vitamin D although how much older was not specified. Only 6% of participants were taking vitamin D supplements, but this finding was not reported by age other than that there was a non-statistically significant difference with those on supplements being on average 35 y/o (versus 33 y/o for non-users). As ages were reported as averages only, it is unclear how many, if any people, were 65 y/o+ although this age range was included amongst the choices for participant ages. When queried about how to increase awareness, 52% of participants suggested leaflets—though where they should be displayed and how to distribute leaflets was not specified-followed by information from GPs and nurses. These findings were not reported by age. The authors stated that “men and older people had particularly low levels of awareness of Vitamin D”, but these findings were not reported by specific age groups [25].

Two small qualitative studies related to vitamin D were recently carried out in the UK. “Target the message” was conducted in the North West of England (Manchester); it assessed vitamin D knowledge and beliefs within a cultural context [26]. 26 participants consisting of white Caucasians and South Asians, age range 21–65 (numbers of older people not specified) participated in focus group discussions. Analysis was conducted by systematic text condensation after use of qualitative data analysis software. South Asian compared with white Caucasian participants were much more knowledgeable about vitamin D including need for sun exposure, dietary sources and its health effects on bones; no participant in either group discussed vitamin D and falls. Although information about preferences for obtaining vitamin D was not an explicitly stated aim of this research, South Asian participants shared that despite knowledge about sun exposure and food sources, they preferred supplementation for improving vitamin D status although few of them were actually taking supplements. Interestingly, white Caucasians were generally averse to supplementation, but had low knowledge about dietary sources with some participants stating a “balanced” diet, or fruits and vegetables, would provide vitamin D [26]. Knowledge about sun exposure practice needed for vitamin D synthesis was very limited amongst both groups.

The “Test me or treat me” study was carried out in a large general practice in East London; purposive sampling was used to ensure participants were from varied ethnic backgrounds and of different ages including 2 groups of people 65 y/o+ [27]. Qualitative data analysis software was used to collect and organise data; framework was used to carry out thematic analysis. This study found that the knowledge and views on vitamin D were, for the most part, similar amongst the different ethnic and age groups. Sources of information, and sources people would use to specifically find information related to vitamin D, were varied and included leaflets, healthcare professionals, the media and the Internet; the Internet was found to be confusing and contradictory with one participant describing it as “a nightmare” [27]. Interestingly, older adults’ knowledge about vitamin D food sources was mostly from their parents and memories of public health campaigns during their childhood such as cod liver oil dosing in schools. In general, levels of accurate knowledge were low, and some participants were confused about vitamin D health effects, thus participants knew vitamin D was important for bones and that sunshine was needed for vitamin D, but not specifically how much sunshine, nor were they aware of the actual effect on bones stating “lack of vitamin D... starts to shrink [them] or something” [27]. No participants were aware of current government recommendations, similar to the findings of the previously discussed Mulhern study [24]. Interestingly, participants stated they would welcome a government based “algorithm” in which one could input data about age, ethnicity, etc., and get specific dietary advice and sun exposure times [27]. The majority of participants from all groups were against universal food fortification. Lastly, the majority of participants would prefer natural sources of vitamin D rather than supplements; indeed, combination calcium and vitamin D supplements were almost universally disliked as illustrated by the following quotes: “they’re great big white tablets, four-a-day—like a horse.” “Disgusting,” “slimy,” and “unpleasant” [27]. The researchers did not find significant differences for most responses by age group other than as stated above.

### 3.3. Older Adult Only Studies

There were fewer older adult only studies in comparison with mixed-population and studies with young adults only identified in the literature search; they were also all smaller size studies. A total of nine older adult only studies were identified with the search terms as specified.

Two of the older adult only studies identified were conducted in Holland; the first used a combination of questionnaires and interviews to identify factors associated with the intention to use vitamin D supplements amongst a random sample of 497 older adults (65 y/o+) [28]. In this study higher belief in self-efficacy and also in risk of being vitamin D deficient and having been given advice to start supplements by a healthcare professional, or participants having been exposed to the Dutch Public Health Council’s advice to use supplements, were associated with intention to start or restart vitamin D supplements [28]. A more recent study used a questionnaire to assess knowledge about vitamin D and its relationship to participants’ vitamin D status amongst 426 older adults (65 y/o+) living in residential care homes [29]. Actual use of supplements was also measured. Only 1/3 of participants were knowledgeable about vitamin D, including government recommendations and dietary sources, but knowledge was positively and strongly correlated with healthy vitamin D status; those with the most knowledge of vitamin D had blood levels of vitamin D approximately twice as high as participants with the least knowledge [29]. In this study some 14% of participants were aware of the association between vitamin D and falls.

A small (*n* = 57) study carried out in Australia explored knowledge about vitamin D in older adults living in a care home with nursing. Participants were recruited from a pool of people being recruited for another larger study assessing vitamin D and falls [30]. Only 4/57 people identified sunlight as a source of vitamin D. Approximately 60% of participants had heard of vitamin D, and of the 11 people who stated they knew the role of vitamin D, 6 knew it “strengthened bones”, 1 person mentioned it strengthened muscles, and 4 others cited generic health benefits. 12 people knew sunlight was a source of vitamin D, however only 4 identified dietary sources including “the pharmacy” “milk and food” and “fruit and vegetables” [30]. Approximately 31% of participants either did not spend time outdoors in the sun, or spent less than 15 min/day.

A cross-sectional questionnaire-based study in Hong Kong (*n* = 648) tested whether “health literacy” regarding vitamin D (for example, knowledge about vitamin D and bone health) and doctor recommendations in older adults led to sunlight exposure behaviour that would be predicted to lead to cutaneous vitamin D synthesis [31]. The study was conducted amongst people 65 y/o+ living in either the community or care homes. The findings supported the investigators hypothesis, that knowledge about vitamin D lead to behaviours that were associated with appropriate sun exposure for cutaneous synthesis.

271 community dwelling older adults (in this study defined as 50 y/o+) participated in an interventional study in Korea to evaluate if an educational intervention to improve knowledge about osteoporosis and vitamin D would lead to increased vitamin D intake [32]. In the 199 participants who completed the intervention, inadequate vitamin D intake was decreased from almost 85% to 66% assessed by a validated questionnaire [32].

Two studies carried out in the USA examined vitamin D practices and subsequent intake within the context of social demographic characteristics and health status. “Food Choices Among Homebound Older Adults” (*n* = 185) was a prospective observational study that used an interviewer administered questionnaire to evaluate food choice motivations and barriers, and 24-h dietary recall to measure dietary vitamin D intake [33]. The majority of participants (82%) had inadequate vitamin D intake (defined as ≤10 μcg/day for people aged 51–70 y/o, and ≤15 μcg/day for people 70 y/o+). People who reported convenience as a food choice motivator, and not being able to shop as a barrier, were less likely to meet recommended vitamin D intakes. In addition, amongst homebound older adults with less education, low price was a positive motivator, and lack of monies a barrier to desired food choices, though this was not reported specifically in relation to vitamin D [33]. The second study measured adherence with calcium/vitamin D supplements as part of a clinical intervention trial with a diverse group of older adults (*n* = 107) [34]. The study aimed to identify characteristics associated with adherence including the effects of ethnicity, socioeconomic status (SES) and health status. Average adherence was high at 77.8% however, after adjustment for study drop-outs, it decreased to 60.7%. A history of fractures, and higher household income and years of education, were all positively associated with adherence. Of note, this study gave combined calcium and vitamin D pills which were large and thus may have contributed to non-adherence; this possibility was not specifically assessed [34].

Age Watch, a UK based charity that funds and carries out research with the aims to promote and sustain evidence-based strategies for positive ageing, conducted a survey measuring vitamin D awareness amongst older adults (*n* = 270) [35]. Survey results indicated that the majority of people were unaware of their vitamin D status, a significant minority of people—38% -- avoided the sun because of concerns about skin cancer even if aware of the need for sunshine to synthesise vitamin D, and some participants were unaware of the health effects of vitamin D.

A small study carried out in the UK (Midlands area) explored factors affecting nutrient intakes, including vitamin D, in older migrant women (age 60 y/o+); a sample of 76 women from various different ethnic backgrounds were purposively selected [36]. Dietary intake was measured by 24-h recall, and semi-structured interviews analysed using thematic analysis. As found in other dietary surveys that have included older adults, such as the National Diet and Nutrition Survey [13] intake of vitamin D did not meet the RNI as set by PHE [8]; average intake was 2.6 μcg/day versus the RNI of 10 μcg/day. However, of the 9 participants who did meet the RNI they did so because they were taking vitamin D supplements. In this study, older, migrant women were more likely to be taking vitamin D supplements if they had been prescribed by a GP.

## 4. Discussion

### 4.1. Knowledge Regarding Vitamin D—Government Recommendations and Health Effects

Older adult vitamin D-related knowledge varied significantly in different geographical regions, although studies carried out in the last several years (for example, [15,19,27,36]) suggest that general knowledge about vitamin D has increased amongst older adults and also the general population. However, the reviewed studies found that knowledge about current government recommended intakes remains low [24,27], and specific and accurate knowledge on the health effects of vitamin D is limited. In particular, participant knowledge of the association of low vitamin D status and falls was identified in only one study [29], suggesting that not only older adults, but also researchers and healthcare professional, do not consider vitamin D and fall risk when discussing vitamin D. This is despite multiple studies identifying low vitamin D as a fall risk factor, and correction of low status with decreased risk of falls or injury [11,38,39,40]. This is an important point as falls and subsequent injury may directly impair mobility and thus affect older adult quality of life, morbidity and mortality. Furthermore, sharing with older adults that adequate vitamin D status reduces fall risk may be a powerful motivator to improving vitamin D levels through dietary intake, sun exposure or supplement use, as fear of falling is common among older adults [41]. This point is also supported by the finding that a history of fractures, the most common fall-related complication, increased the likelihood of older adults using vitamin D supplements [34].

### 4.2. Knowledge Regarding Vitamin D—Dietary Sources

The identified studies also indicate considerable confusion and inaccuracy about vitamin D dietary sources amongst all participants including older adults. Although in most studies a significant proportion of older adults could identify some dietary vitamin D sources, and in selected studies older people were more knowledgeable about food sources of vitamin D compared with younger participants (for example [15,16,17,18]), a significant number of older people misidentified certain foods as good sources of vitamin D, or assumed that a “balanced diet” would ensure adequate intake of vitamin D [26]. The finding in one large study conducted in the USA that the majority of older adults surveyed felt that their diet and vitamins provide adequate vitamin D, whilst multiple national dietary surveys have consistently documented both low intakes and low blood levels in a significant minority of older adults, also suggests misinformation about vitamin D containing foods [20]. Low vitamin D status is a persistent problem amongst older adults, and in order to enable older adults to make informed choices to improve dietary intake, healthcare professionals and public health bodies need to develop dietary guidelines that clearly identify vitamin D rich foods, and effective communication strategies for disseminating these guidelines specifically targeted towards older people.

### 4.3. Knowledge Regarding Vitamin D—Sun Exposure

Although many study participants were aware that sun-exposure was necessary for cutaneous vitamin D synthesis, there was confusion amongst older adults about the balance of benefits and risks from sun exposure for vitamin D synthesis versus increased risk of skin cancer; furthermore, lack of knowledge about time of day, and duration of time needed for sun-induced vitamin D synthesis, was demonstrated in every identified study that included questions about sun exposure/behaviours. Inadequate knowledge about how much sun exposure is required may arise in part because health bodies and professionals themselves cannot give precise recommendations. For example, NICE guideline 34 (NG34) “Sunlight exposure: risks and benefits” recommends “short periods” to allow for cutaneous vitamin D synthesis but without burning [42]. Similarly, NHS Choices also recommends exposure of the forearms, hands and lower legs for “short periods” (NHS website accessed May 2018), and the British Association of Dermatologists’ “Consensus vitamin D position statement” (2010) on sunlight and vitamin D synthesis recommends “little and often”. The presented evidence in this review suggests neither the public nor healthcare professionals know what these terms really mean. There is available evidence about specific amounts of time needed for cutaneous vitamin D production without burning; 10–30 min in the sun between 11:00 a.m. and 3:00 p.m., without sunscreen, on average 3–5 times a week (in the appropriate season) [43]. However, duration of needed exposure is affected by cloud cover, skin type, pollution, season, age, and ethnicity [5,6,43] and this makes it difficult to give precise “general” recommendations. This does not negate the need for clear public health guidelines, and clear advice from healthcare professionals, to enable older adults to make informed choices about sun exposure to improve vitamin D status, especially as amongst some older adults there is aversion to the use of supplements. The suggestion from one study participant for a government algorithm [27], presumably available online, that could be used to calculate the duration of needed sun-exposure after the entry of variables such as gender and age, deserves consideration. There is research that suggests older adults will consider using technology to improve communication and health [44,45], and thus whether technological tools could improve vitamin D nutritional education and interventions to improve status in older adults warrants exploration.

### 4.4. Knowledge Regarding Vitamin D—Information Sources and Increasing Awareness

In the majority of studies older adults identified healthcare professionals, in particular their GPs, as primary and preferred information sources, and in several studies advice from a GP to use vitamin D supplements was associated both with increased use of supplements and also better vitamin D status [26,28,36]. However, a significant number of older adults also indicated that healthcare professionals did not discuss vitamin D with them, or only did so to a limited extent [17,19]. This suggests that an opportunity is being missed to effectively communicate vitamin D supplement recommendations by healthcare professionals identified as trusted sources by older adults. It is possible that GPs and other primary healthcare providers do not recommend vitamin D to all their older adult patients because of GP’s uncertainty about the need for supplementation [46]; indeed, research has found that uncertainty regarding guidelines may lead to clinical inertia on the part of physicians [47]. This suggests that the medical education and research community, and government public health bodies, need to clearly communicate to healthcare professionals the evidence base and rationale for promoting vitamin D to their patients. Furthermore, it needs to be made clear to older adults that vitamin D supplements *alone* are recommended unless dietary calcium intake is inadequate; use of combined calcium and vitamin pills may decrease compliance, and the levels of vitamin D in multivitamins may be inadequate [16]. Adherence with supplementation might be improved with consistent recommendations for vitamin D only pills as suggested by guidelines. Increasing older peoples’ awareness of vitamin D should encompass multiple strategies at different levels. Older adults identified various forms of communication including leaflets, posters, newspapers, and the radio as useful for obtaining knowledge about vitamin D, and all of these could be part of an implementation strategy to increase awareness and knowledge related to vitamin D. In addition, preferred communication means should be continually re-evaluated as adults in the current technological environment may have different communication preferences as they progress into older age.

### 4.5. Knowledge Regarding Vitamin D—Impact on Behaviour

Interestingly, improved knowledge amongst study participants—whether regarding dietary sources of vitamin D, need for supplementation, or sun-exposure—did not always correlate with associated behaviours that would lead to improvements in vitamin D intake or status [15,21,25,26,27]. However, this may be affected by the knowledge source, and the exact information about vitamin D being communicated, as two studies in different countries found that improved knowledge about vitamin D did result in increased intake, and in the one study that measured serum 25(OH)cholecalciferol levels supplement use was associated with improved vitamin D sufficiency [31,32]. Public health officials and healthcare professionals may need to consider what strategies would be most effective in increasing knowledge and awareness of vitamin D to improve older adult vitamin D status; discussions with older people exploring alternatives should inform the development and implementation of such strategies.

### 4.6. Older Adult Socio-Demographic Factors Affecting Vitamin D Knowledge, Beliefs and Practices

This review found that the impact of socio-demographic factors on vitamin D knowledge and practices varied between different locations. The trend for lower socio-economic status and education to be associated with lower levels of knowledge and vitamin D intake, coupled with barriers such as the ability to shop and cost to access vitamin D through foods or supplements, at least in the USA, suggests that effective guidelines and implementation strategies need to adjust and accommodate for vulnerable older adult populations in order to be effective. In particular, in homebound, and possibly in older adults living in care homes, different methods of information dissemination and availability of foods and/or supplements should be considered to improve intake and thus vitamin D sufficiency.

### 4.7. Older Adult Preferences for Obtaining and Sustaining Healthy Vitamin D Status

There was considerable variation in responses regarding how older adults would prefer to improve their vitamin D status, with evidence of cultural, geographic and age-related effects on preferences. The finding that vitamin D supplements combined with calcium were “disgusting” [27] is in accordance with findings reported in NICE Public Health Guideline 56 that older adults dislike combination vitamin D and calcium supplements [6]. Indeed, as previously discussed, PHE recommends vitamin D alone supplements for prevention of deficiency, and does not stipulate combination supplements unless there is evidence of inadequate dietary calcium [8].

However, the evidence regarding older adult preferences for obtaining vitamin D is limited; no studies were identified that had the specific aim of exploring older adult preferences for obtaining vitamin D and ensuring maintenance of adequate status. This is an essential research question in order to be able to develop effective older adult-centred strategies to decrease the risk of vitamin D deficiency.

## 5. Conclusions

A clear understanding of preferences, integrated into both public health guidelines and implementation strategies, is key not only to decreasing the risk of vitamin D deficiency but also to enabling autonomy in older adults. The importance of autonomy and recognition as guiding principles in sustaining health and successful ageing applies to most people, however, it is particularly evocative in the case of older adults who may be patronised and deprived of autonomy by the healthcare community [48]. NICE guideline number 32, “Older People: Independence and mental wellbeing”, states that “People using services have the right to be involved in discussions and make informed decisions about their care” [49]. The evidence from studies in this review suggests that available information regarding vitamin D needs to be more clearly communicated and discussed with older people, and that further research on older adult preferences for obtaining and sustaining healthy vitamin D levels is required, so that older adults are indeed in the position to make informed decisions about their care.

## Figures and Tables

**Table 1:** Characteristics and key findings of reviewed studies.

Study	Total *n*	N Older Adults/% (% of Total *n*)	Study Design	Country	Key Findings	Remarks
**Deschasaux et al. 2016** [15]	59,273	22,728/38%	Quantitative online survey; blood samples analysed for 25(OH)cholecalciferol in a subset of participants	France	Older adults had the highest vitamin D awareness compared with all other ages; awareness did not always correlate with vitamin D sufficiency	Older adults were defined as ≥55 y/o; no further age breakdown was provided
**Black et al. 2016** [16]	12,513	4179/33%	Quantitative survey-based interviews Dietary assessment by 24-h recall Blood samples analysed for 25(OH)cholecalciferol in a subset of participants	Australia	Older adults were more likely to be using vitamin D supplements or multivitamins containing vitamin D and this corresponded with increased blood levels of 25(OH)cholecalciferol	Only 30% of older adults were using vitamin D containing supplements; and only 4% were on vitamin D alone supplements as recommended
**Vu et al. 2010** [17]	2876	318/9.5%	Quantitative cross-sectional online survey	Australia	Vitamin D knowledge was higher amongst older adults but most participants mis-identified food sources, and there was also confusion about sun exposure times	Only 10% of older adults were worried about their vitamin D status
**Janda et al. 2010** [18]	2000	1319/66%	Quantitative cross-sectional phone survey	Australia	Vitamin D knowledge was higher amongst older adults; less than 10% participants knew sun exposure times needed for vitamin D	Only age differentiation provided was whether people were younger or older than 40 y/o
**Bonevski et al. 2013** [19]	52	18/35%	Qualitative Used consolidated criteria for reporting qualitative findings and two health behaviour models for study analysis	Australia	Although the majority of participants had heard of vitamin D few could name its health effects nor needed sun exposure time for cutaneous synthesis	Participants were much more aware, and concerned about, avoidance of the sun and skin cancer risks
**Holman et al. 2017** [20]	4127	1460/35.4%	Cross-sectional quantitative online survey retrospective	USA	Older adults were more likely to agree with the statement that they could meet vitamin D recommended intakes through diet and vitamins	Older adults tended to not feel that their sun-protection habits increased their risk of vitamin D deficiency
**Kung et al. 2006** [21]	547	172/31.5%	Cross-sectional quantitative online survey	Hong Kong, China	Conversely compared with other studies, as age increased vitamin D-related knowledge decreased; amongst all participants knowledge about foods sources was low	This was an all-female study Older women preferred being outdoors, and knowledge of sunlight-induced vitamin D did not correlate with sun exposure
**Al Bathi et al. 2012** [22]	200	52/26%	Cross-sectional quantitative survey administered questionnaire Blood samples analysed for 25(OH)cholecalciferol	Kuwait	Approximately half of all participants were unaware of the association between musculoskeletal health and vitamin D despite participants being selected from a group of women being treated for deficiency	This was an all-woman study Average levels of 25(OH)cholecalciferol for women 70 y/o+ met criteria for deficiency
**Aljefree et al. 2017** [23]	22	18/82%	Qualitative investigation conducted by semi-structured interviews; analysed using thematic analysis	Saudi Arabia	Overall good knowledge about vitamin D including its health effects and association with sunlight; low knowledge about dietary sources	This was an all woman study; older adults were defined as 49 y/o+; high temperatures were a disincentive to being outdoors in the sun
**Mulhern et al. 2017** [24]	1320	Approximately 660/50%	Cross-sectional anonymous online survey	UK/primarily Northern Ireland	Majority study participants had heard of vitamin D, but less than 10% were aware of new UK recommendations for vitamin D intake	Age range included older adults, but further age categorisation was not provided
**Alemu et al. 2012** [25]	221	Unavailable	Quantitative cross-sectional questionnaire-based study	UK	Older participants had lower awareness of vitamin D; only 6% participants were on supplements but this was not reported by age	Average age was 35 y/o; age range included older adults but further age categorisation was not provided
**Webb et al. 2016** [26]	26	Unavailable	Qualitative focus-group based investigation analysed by systemic text condensation after qualitative data analysis software	UK	South Asian participants were more knowledgeable about vitamin D including dietary sources compared with white participants. White participants had low levels of knowledge about dietary sources	South Asian participants preferred supplements for obtaining vitamin D whereas white participants were averse to supplementation
**Kotta et al. 2015** [27]	58	21/36%	Qualitative focus groups; data collected by qualitative data analysis software and analysed using framework thematic analysis	UK	Levels of accurate vitamin D knowledge were low and not differentiated by age group; older adult information sources were cited as childhood public health campaigns and parents. No participants were aware of current DoH recommendations	Both older adults in the community and care homes were included. Participants noted the Internet as an information source was a “nightmare” and called combination vitamin D/calcium pills “disgusting.”
**Engels et al. 2001** [28]	497	497/100%	Cross-sectional quantitative interviewer delivered questionnaires	The Netherlands	Higher belief in self-efficacy associated with the belief of being at risk for vitamin D deficiency predicted vitamin D supplement use	Recommendations from healthcare professionals increased likelihood of use or intention to use supplements. Actual use of supplements or blood measurements were not conducted
**Oudshoorn et al. 2012** [29]	426	426/100%	Cross-sectional quantitative interviewer administered questionnaire Blood samples analysed for 25(OH)cholecalciferol	The Netherlands	Only 1/3 of participants were knowledgeable about vitamin D, but knowledge was strongly positively correlated with supplement use	This study measured actual supplement use and blood levels of 25(OH)cholecalciferol
**Durvasula et al 2010** [30]	57	57/100%	Quantitative validated questionnaire and qualitative semi-structured interviews with the inclusion of open-ended questions analysed by thematic analysis	Australia	The majority of participants had heard of vitamin D (59.6%) and of those 20% had some idea of its role in health including healthy bones and muscles; knowledge of dietary sources was low	Although the majority of respondents felt they had adequate sun exposure this was not actually measured to see if perceptions correlated with actual exposure
**Leung et al. 2014** [31]	648	648/100%	Cross-sectional quantitative questionnaire	Hong Kong, China	Knowledge about vitamin D and its health affects specifically lead to behaviours that were associated with appropriate sun exposure for cutaneous synthesis; actual knowledge levels amongst participants were low	No actual observation of sun-related behaviours was made nor blood measurements taken to see if behaviours were associated with vitamin D sufficiency
**Park et al. 2017** [32]	271	271/100%	Prospective interventional trial	Korea	Nutrition education including informing participants they were not meeting recommended intakes of vitamin D led to a decrease in inadequate vitamin D intake from 84.4% to 65.8%	No control group somewhat limits the interpretation of findings, and follow up was only for three months
**Locher et al. 2009** [33]	185	185/1005	Qualitative observational design with standard interview techniques coupled with a questionnaire and quantitative dietary assessment by 24-h dietary recalls × 3	USA	The majority of participants had inadequate vitamin D intake at baseline; convenience as a food choice motivator and not being able to shop were strongly associated with low vitamin D intake.	The study was conducted amongst homebound participants and thus these motivators and barriers may be very specific to this group; vitamin D status was not measured to see if low intake correlated with vitamin D deficiency
**Unson et al. 2006** [34]	107	107/100%	Interventional clinical trial	USA	Lower socioeconomic status/household income and education was associated with lower adherence with calcium/vitamin D supplement use, whilst a history of fractures predicted adherence with supplement use	This study gave combined calcium and vitamin D pills which were large and thus may have contributed to non-adherence; this possibility was not specifically assessed
**Age Watch UK 2017** [35]	270	270/1005	Quantitative cross-sectional online survey; available on the charity’s website	UK	The majority of participants were unaware of their vitamin D status, and some 38% avoided sunlight despite recognising its role in vitamin D synthesis out of fears about skin cancer	This survey was not published in a peer-reviewed journal.
**Castaneda-Gameros et al. 2017** [36]	76	76/100%	Qualitative semi-structured interviews analysed using thematic analysis plus quantitative dietary assessment through use of 24-h dietary recall	UK	The majority of participants did not meet UK vitamin D dietary recommendations; women on supplements were more likely to meet recommended intakes, and GP prescription was associated with increased use of supplements	Supplement intakes were self-reported, and there was no blood work analysis to assess for levels of vitamin D sufficiency vs deficiency
